# *Nps*‐Expressing Neurons Receive Extensive Input From Auditory Brainstem Nuclei

**DOI:** 10.1002/cne.70156

**Published:** 2026-04-09

**Authors:** Richie Zhang, Silvia Gasparini, Joel C. Geerling

**Affiliations:** ^1^ Department of Neurology and Iowa Neuroscience Institute University of Iowa Iowa City Iowa USA

**Keywords:** cochlear nucleus, inferior colliculus, neuropeptide S, NPS, parabrachial nucleus, reticular formation, superior olivary complex

## Abstract

Neurons that express *Nps* send output to brain regions implicated in circadian function and threat responses, but less is known about the afferent control of these neurons. In this study, we used a sensitive retrograde tracer, cholera toxin beta subunit (CTb), to identify afferents to the rostral–lateral parabrachial region that contains the main concentration of *Nps*‐expressing neurons. We then used Cre‐dependent rabies retrograde tracing in *Nps*‐2A‐Cre mice to identify inputs to *Nps*‐expressing neurons within this region. These neurons receive heavy input from auditory brainstem structures, including the inferior colliculus, ventral nucleus of the lateral lemniscus, superior olivary complex, and cochlear nucleus. Due to a discrepancy between sparse rabies and prominent CTb labeling extending from the ipsilateral insular to auditory areas of the cerebral cortex, we also performed anterograde labeling and found many close contacts between cortical boutons and *Nps‐*expressing neurons. These findings suggest an unexpected role for auditory information in controlling the activity of *Nps‐*expressing neurons and add to existing evidence suggesting that rabies is relatively insensitive to retrograde labeling of cortical afferents.

## Graphical abstract

*Nps*‐expressing neurons receive input from auditory brainstem nuclei, with additional afferents from the reticular formation, midbrain, hypothalamus, and extended amygdala. While rabies retrograde labeling in the cerebral cortex was sparse, cholera toxin beta (CTb) revealed copious input, and anterograde tracing identified many axons and boutons surrounding parabrachial *Nps*‐expressing neurons.

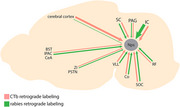

## 1 Introduction

The parabrachial nucleus (PB) integrates interoceptive sensory inputs from the hindbrain with descending modulatory inputs from the forebrain to shape behavioral and physiological responses that maintain homeostasis (Carter et al. 2015; Gasparini et al. 2019; Geerling et al. 2016; Grady et al. 2025; Kaur et al. 2017; Mu et al. 2017; Nakamura and Morrison 2008, 2010; Palmiter 2018; Shin et al. 2011).

Neurons in this region have a variety of different molecular identities and connectivity patterns (Karthik et al. 2022; Pauli et al. 2022). One subset of neurons at the edge of this region expresses *Nps*, which encodes neuropeptide S (NPS; Adori et al. 2015; Clark et al. 2011; Huang et al. 2022; Xu et al. 2004). Pharmacological studies have shown the effects of NPS on locomotion, anxiety, arousal, and food intake (Leonard et al. 2008; Peng et al. 2010; Smith et al. 2006; Xu et al. 2004). Its receptor (*NPSR1*) has been implicated in anxiety, asthma, and endometriosis (Domschke et al. 2011; Laitinen et al. 2004; Tapmeier et al. 2021), and a rare gain‐of‐function *NPSR1* variant reduces sleep (Xing et al. 2019).

Previously, we mapped the output projections of *Nps*‐expressing neurons in the PB region. These neurons target a variety of brain regions, prominently including the paraventricular nucleus of the thalamus, bed nucleus of the stria terminalis, hypothalamus, and tectal longitudinal column (Zhang et al. 2024). Subsequent studies suggested that *Nps*‐expressing neurons in the PB region may promote wakefulness, while those in the central pontine gray may promote sleep (Angelakos et al. 2023; Xing et al. 2024).

The primary concentration of *Nps*‐expressing neurons is found in the rostral–lateral PB, immediately dorsal to the Kölliker–Fuse nucleus, and extends rostrally alongside the lateral lemniscus in the semilunar nucleus (Huang et al. 2022). This population is centered in a subregion described in rats as the “extreme lateral” subnucleus (Fulwiler and Saper 1984). Unlike other PB subnuclei, the extreme lateral subnucleus does not appear to receive input from viscerosensory neurons in the nucleus of the solitary tract (Herbert et al. 1990). The sources of input to this rostral–lateral subregion remain unclear.

In this study, we characterized the brain‐wide afferents to this rostral–lateral parabrachial region using conventional retrograde axonal tracing. Next, we identified afferents specifically to *Nps*‐expressing neurons in this region using Cre‐dependent rabies retrograde tracing in *Nps*‐2A‐Cre mice. In addition, due to a discrepancy between conventional and rabies retrograde labeling in the cerebral cortex, we performed anterograde, presynaptic labeling to investigate direct cortical input. Overall, our findings revealed an unexpected input pattern that suggests a significant role for auditory information in controlling the activity of NPS neurons.

## 2 Materials and Methods

### 2.1 Mice

All mice were group‐housed in a temperature‐ and humidity‐controlled room on a 12/12 h light/dark cycle with ad libitum access to water and standard rodent chow. Overall, we used 13 female and male mice ranging in age from 9 to 12 weeks (17–29 g body weight). We used *Nps*‐2A‐Cre and *Nps*‐2A‐Cre;R26‐LSL‐L10GFP Cre‐reporter mice, with detailed information provided in Table 1. All Cre‐driver and reporter mice were hemizygous and maintained on a C57BL/6J background. All experiments were conducted in accordance with the guidelines of the Institutional Animal Care and Use Committee at the University of Iowa.

**TABLE 1:** Cre‐driver and Cre‐reporter mice.

Strain	References	Source information	Key gene
*Nps*‐2A‐Cre	Huang et al. (2022)	Available from The Jackson Laboratory (Jax #038113)	2A‐Cre inserted immediately after terminal exon in endogenous neuropeptide S gene
R26‐LSL‐L10GFP reporter	Krashes et al. (2014)	Available from originating investigators.	Floxed transcription STOP cassette followed by EGFP/Rpl10 fusion reporter gene under control of the CAG promoter targeted to the Gt(ROSA)26Sor locus

### 2.2 Stereotaxic Injections

Mice were anesthetized with isoflurane (0.5%–2.0%) and placed in a stereotaxic frame (Kopf 1900 or 940). We made a midline incision and retracted the skin to expose the skull over the brain. Through a pulled glass micropipette (20–30 µm inner diameter), we targeted the rostral–lateral parabrachial region in each retrograde case with coordinates: 4.7‐mm caudal to bregma, 1.7‐mm right of midline, and 3.8‐mm deep to bregma. Each injection was made over a 5‐min period using picoliter air puffs through a solenoid valve (Clippard EV 24V DC) pulsed by a Grass stimulator. The pipette was left in place for an additional 3 min and then withdrawn slowly before closing the skin with Vetbond (3M). Meloxicam (1 mg/1 kg) was provided for postoperative analgesia.

For retrograde tracing, we unilaterally injected 30 nL of cholera toxin beta subunit (CTb; 0.1% in distilled water; List Biological Laboratories) in *n* = 5 *Nps*‐2A‐Cre;R26‐LSL‐L10GFP Cre‐reporter mice (Cases 7209, 7210, 7213, 7216, and 7220). We excluded one case (7210) from further analysis because the injection site was tiny and did not produce any retrograde labeling.

For Cre‐dependent retrograde tracing with modified rabies, we used five *Nps*‐2A‐Cre mice without a Cre reporter (Cases 6269, 6270, 6271, 6272, and 6273). First, we performed bilateral injections delivering 60 nL of a 1:1 mixture of two “helper viruses” supplying the avian receptor required for entry of EnvA‐pseudotyped rabies (AAV8‐EF1a‐TVA‐mCherry; Table 2) and the glycoprotein required for G‐deleted rabies retrograde entry into non‐TVA‐expressing afferents (AAV8‐CAG‐FLEX‐RabiesG; Table 2). After 14 weeks, we injected 200 nL of EnvA‐pseudotyped, G‐deleted rabies‐eGFP and then perfused each mouse 8 days later.

**TABLE 2:** Antisera used in this study.

**Antigen**	**Immunogen description**	**Source, host species, RRID**	**Concentration**
Cholera toxin b subunit (CTb)	Cholera toxin b subunit	List Biological Laboratories, goat polyclonal, Cat.# 703, Lot#: 7032A10; RRID:AB_10013220	1:10,000
Forkhead box protein P2 (FoxP2)	Recombinant human FOXP2 isoform 1 Ala640‐Glu715	R&D Systems, sheep polyclonal, cat. #AF5647, RRID: AB_2107133	1:3000
Green fluorescent protein (GFP)	Full length green fluorescent protein from the jellyfish *Aequorea victoria*	Thermo Fisher Scientific, chicken polyclonal, Cat.# A10262, Lot#: 1972783, RRID:AB_2534023	1:3000
mCherry	Full length mCherry fluorescent protein	Life Sciences, rat monoclonal, Cat.# M11217, Lot#: R1240561, RRID:AB_2536611	1:2000
Tyrosine hydroxylase (TH)	Denatured TH from rat pheochromocytoma in rabbit host	Millipore, rabbit polyclonal, Cat.# AB152, Lot#: 400383, RRID:AB_390204	1:10,000

For non‐Cre‐dependent anterograde tracing from the cerebral cortex, we unilaterally injected AAV8‐hEf1a‐synaptophysin‐mCherry (Table 2) in three *Nps*‐2A‐Cre;R26‐LSL‐L10GFP Cre‐reporter mice (Cases 9327, 9328, and 9329). We injected 400 nL total per mouse at two separate levels (2.25 and 2.5 mm caudal to bregma, both 4.0 mm right of midline) at two depth coordinates each (3.25 and 3.75 mm deep to bregma). Each of these four 100 nL injections was made over a 2‐min period. The pipette was left in place at the ventral coordinate for an additional 2 min after injection, withdrawn slowly to the dorsal coordinate, left in place for an additional 5 min after injection, and then withdrawn slowly from the brain.

### 2.3 Perfusion and Tissue Sections

All mice were anesthetized with a mixture of ketamine‐xylazine (ip 150–15 mg/kg, dissolved in sterile 0.9% saline) and then perfused transcardially with phosphate‐buffered saline (PBS), followed by 10% formalin‐PBS (SF100‐20, Thermo Fisher Scientific). After perfusion, the brains were removed and fixed overnight in 10% formalin‐PBS. We sectioned each brain into 40‐µm‐thick coronal slices using a freezing microtome and collected tissue sections into separate, 1‐in‐3 series. Sections were stored in cryoprotectant solution at −30°C until further processing.

### 2.4 Immunofluorescence Labeling

We removed the tissue sections from the cryoprotectant and rinsed them in PBS before loading them into a primary antibody solution (Table 3). Antisera were added to a PBS solution of 0.25% Triton X‐100 (BP151‐500, Thermo Fisher Scientific) and 2% normal donkey serum (NDS, 017‐000‐121, Jackson ImmunoResearch), plus 0.05% sodium azide (14314, Alfa Aesar) as a preservative (PBT‐NDS‐azide). After incubating tissue sections overnight at room temperature on a tissue shaker, the sections were washed three times in PBS and incubated at room temperature in PBT–NDS–azide solution containing species‐specific donkey secondary antibodies. These secondary antibodies were conjugated to Cy3, Cy5, or Alexa Fluor 488 (Jackson ImmunoResearch Catalog numbers 705‐165‐147, 703‐545‐155, 711‐175‐152; each diluted 1:1000 or 1:500). After 2 h in the secondary antibody solution, tissue sections were washed three times in PBS, mounted on glass slides (#2575‐plus; Brain Research Laboratories), and coverslipped using Vectashield with DAPI (Vector Labs). Slides were stored in slide folders at 4°C until imaging.

**TABLE 3:** Viral vectors.

Injected tracer	Origin
EnvA‐G‐deleted rabies‐eGFP, Titer: 2.86 × 10^8^	Salk Viral Vector Core, RRID:Addgene_32635
AAV8‐hEf1a‐Synaptophysin‐mCherry, Titer: 2.3 × 10^13^	Dr Rachel Neve at the Massachusetts Institute of Technology McGovern Institute for Brain Research Viral Vector Core
AAV8‐CAG‐FLEX‐rabiesG, Titer: 9.2 × 10^12^	Stanford Vector Core, Lot: 6751, RRID:Addgene_48333
AAV8‐Ef1a‐FLEX‐TVA‐mCherry, Titer: 6 × 10^12^	UNC Vector Core; Lot: AV5006b, RRID:Addgene_38044

### 2.5 Imaging, Analysis, and Figures

All slides were scanned using a VS120 microscope (Olympus Corporation). We first acquired a 2× overview scan and then used a 10× objective to scan all tissue sections. We also acquired confocal images of anterogradely labeled presynaptic boutons using a 60× oil objective on a STELLARIS confocal microscope (Leica Microsystems).

In every rabies retrograde tracing case, we used QuPath (Bankhead et al. 2017) to plot all rabies‐eGFP‐ and TVA‐mCherry‐labeled cells. We counted every cell that contained in‐focus labeling throughout every tissue section at every level of every brain. Counts were aggregated by brain region, divided by the total number of rabies‐eGFP neurons labeled in each case, and divided by the total number of injection site “starter cells” (injection site neurons containing both rabies‐eGFP and TVA‐mCherry) in Microsoft Excel. These data were used to generate graphs in GraphPad Prism.

To prepare images for figures, we first used LAS X Office (Leica Microsystems) or QuPath to crop full‐resolution images and Adobe Photoshop to adjust brightness and contrast. We used Adobe Illustrator to arrange images, add lettering for all figure layouts, make drawings, and plot cells for the brain‐wide illustration of labeling in Case 6273. Scale bars were traced in Illustrator atop calibrated lines from QuPath to produce clean white or black lines in each figure.

### 2.6 Nomenclature

For neuroanatomical structures and cell populations, where possible, we use and refer to nomenclature defined in peer‐reviewed neuroanatomical literature. In some instances, we use or refer to nomenclature derived from rodent brain atlases (Dong 2008; Paxinos and Franklin 2013; Paxinos and Watson 2007; Swanson 1992).

## 3 Results

### 3.1 CTb Cases

**FIGURE 1:**
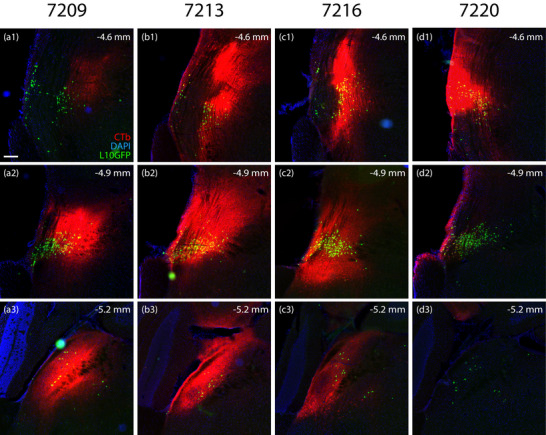
Cholera toxin b (CTb) injection sites in *Nps*‐2A‐Cre;R26‐LSL‐L10GFP mice. Each CTb injection site is shown in a series of rostral (top row) to caudal (bottom row) levels in each of four cases: (a1–a3) 7209, (b1–b3) 7213, (c1–c3) 7216, and (d1–d3) 7220. The approximate level caudal to bregma is shown in the upper‐right corner of each panel. CTb is shown in red, L10GFP in green, and DAPI in blue. The scale bar in (a1) is 200 µm and applies to all panels.

We analyzed retrograde labeling after unilateral CTb injection into the parabrachial region in four *Nps*‐2A‐Cre;R26‐LSL‐L10GFP mice. The injection site varied between cases (Figure 1). In Case 7209, the center of the injection site overlapped the dorsomedial aspect of the main cluster of NPS neurons in the extreme lateral parabrachial nucleus and extended caudally into the middle of the parabrachial nucleus. In Case 7213, the center of the injection site overlapped the main cluster of NPS neurons and extended dorsally into the superior lateral parabrachial nucleus. In Case 7216, the center of the injection site overlapped the main cluster of NPS neurons and extended both ventrally into the Kölliker–Fuse nucleus and rostrally into the semilunar nucleus. In Case 7220, the injection site was centered rostrally, overlapping the dorsolateral cluster of NPS neurons in the semilunar nucleus. Each injection produced a different pattern of retrograde labeling (Table 4), as described below.

**TABLE 4:** Brain‐wide ratings of CTb‐labeled neurons for all cases.

	7209	7213	7216	7220
Motor cortex	+	+	+	−
Primary somatosensory area	+	+	+	−
Medial prefrontal cortex	+++	+++	+++	−
Deep orbital‐medial cortex	++++	+++	+++	−
Orbital cortex	+++	+++	+++	−
Insular‐perirhinal/entorhinal cortex	+++	++	++	−
Auditory cortex	++++	+++	++++	++++
Bed nucleus of the stria terminalis	+++	−	+	−
Preoptic area	+++	++	+++	−
Anterior amygdalar area and cortical amygdalar area	++++	+++	+++	−
Substantia innominata and interstitial nucleus of the posterior limb of the anterior commissure	+++	−	−	−
Central nucleus of the amygdala	++++	−	−	−
Paraventricular hypothalamic nucleus	+++	+	+++	+
Dorsomedial hypothalamic nucleus	+++	++	++	−
Ventromedial hypothalamic nucleus	+	++	+++	−
Lateral hypothalamic area	+++	++	++	+
Parasubthalamic nucleus	++++	+++	+++	+
Zona incerta	++	++	−	+
Pretectal nucleus	+	++	++	+
Peri‐medial geniculate nucleus	+++	++	++	++
Paraventricular nucleus of the thalamus	−	−	−	−
Hippocampus	−	−	−	−
Midbrain reticular formation	++++	+++	++++	−
Superior colliculus	+++	+++	++++	++++
Periaqueductal gray	++++	++++	++++	++++
Inferior colliculus	+++	+++	+++	++++
Dorsal nucleus of the lateral lemniscus	+	++	++	+++
Ventral nucleus of the lateral lemniscus	+	+++	+++	++++
Cochlear nucleus	+	+++	+++	++++
Superior olivary complex	+	++++	++++	++++
Principal sensory trigeminal nucleus	++	−	−	−
Pontine reticular formation	++++	++	+++	−
Medullary reticular formation	++++	++	++++	−
Nucleus of the solitary tract and area postrema	++++	+++	++++	+
Spinal trigeminal nucleus	+++	++	+++	+
Vestibular nucleus	+	+	+	−

*Note:* ++++ = 50+ neurons, +++ = 25+ neurons, ++ = 10+ neurons, + = < 10 neurons, − = 0 neurons.

#### 3.1.1 Cerebral Cortex

**FIGURE 2:**
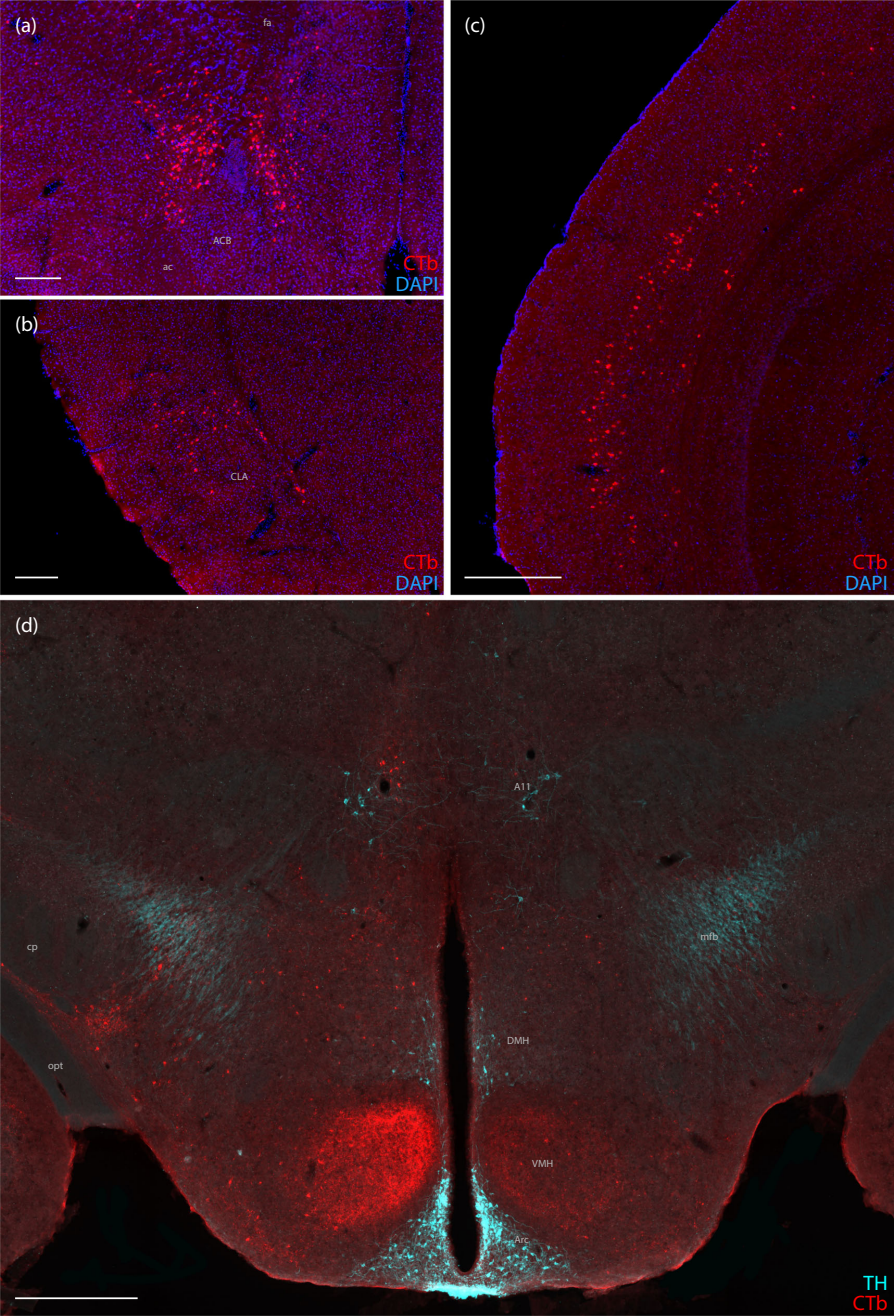
CTb labeling in the cerebral cortex and hypothalamus. CTb‐labeled neurons (red) in the (a) deep orbitomedial prefrontal cortex, rostral dorsal to the nucleus accumbens (ACB; Case 7209), (b) insular cortex near the claustrum (CLA) and embedded in the external capsule (Case 7209), (c) auditory cortex (Case 7220), and (d) hypothalamus, including anterograde axonal labeling within the ventromedial hypothalamic nucleus (VMH; Case 7213). CTb‐labeled neurons can also be seen above the A11 group, with tyrosine hydroxylase (TH) immunoreactivity shown in ice blue. The scale bars in (a) and (b) are 250 µm, the scale bar in (c) is 400 µm, and the scale bar in (d) is 500 µm. ac, anterior commissure; Arc, arcuate hypothalamic nucleus; cp, cerebral peduncle; fa, anterior forceps of the corpus callosum; DMH, dorsomedial hypothalamic nucleus; mfb, medial forebrain bundle; opt, optic tract.

The orbital cortex bilaterally contained a modest number of retrogradely labeled neurons in all cases except 7220. These cases also had many CTb‐labeled neurons within the infralimbic and prelimbic areas of the medial prefrontal cortex. These CTb‐labeled neurons were present bilaterally, but more on the ipsilateral side in every case. Caudally, this retrogradely labeled population extended ventrally to surround the rostraldorsal pole of the nucleus accumbens (Figure 2a), occupying a region of the deep orbitomedial prefrontal cortex that has been described as “area lambda” (Souza et al. 2022). Each of these cases also had a few CTb‐labeled neurons in the ipsilateral primary and secondary motor cortices, and this sparse labeling extended caudally through the secondary motor and primary somatosensory cortex.

Most CTb‐labeled neurons in the cerebral cortex were located in the insular area and contiguous cortical areas caudal and dorsal to it, along and dorsal to the rhinal fissure. In the insular area, this population was split into Layer 5 and a deeper layer located immediately dorsal to the claustrum and embedded within the external capsule (Figure 2b), as described previously (Grady et al. 2020). A similarly split pattern of retrograde labeling extended caudally into Layers 5 and 6b/7 of the perirhinal, ectorhinal, and temporal association areas and then continued dorsally into the auditory cortex. In contrast to the other three cases, CTb retrograde labeling in Case 7220 was shifted caudally and dorsally, with extensive labeling in the auditory cortex yet no labeling in prefrontal or insular areas (Figure 2c).

#### 3.1.2 Bed Nucleus of the Stria Terminalis (BST) and Amygdala

**FIGURE 3:**
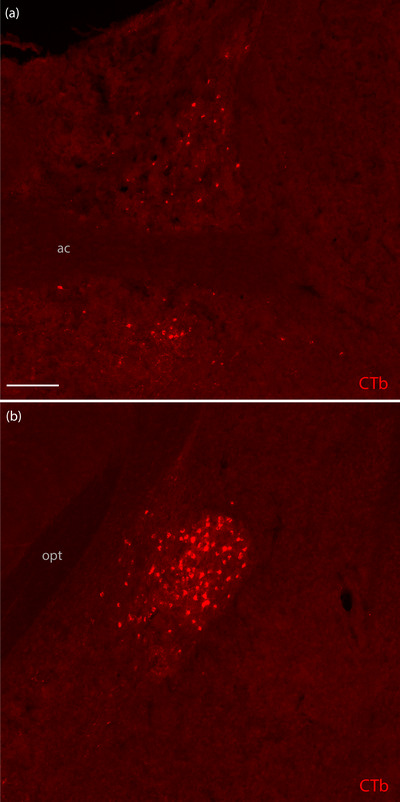
CTb labeling in the bed nucleus of the stria terminalis and amygdala. CTb‐labeled neurons (red) in the (a) bed nucleus of the stria terminalis and (b) central nucleus of the amygdala in Case 7209. The scale bar in (a) is 200 µm and applies to (b). ac, anterior commissure; opt, optic tract.

While Case 7209 had a substantial number of CTb‐labeled neurons (Figure 3a), primarily in the oval BST subnucleus, 7216 had only a few labeled neurons in this region, and Cases 7213 and 7220 had none. Cases 7209, 7213, and 7216 had a moderate number of CTb‐labeled neurons in the interstitial nucleus of the posterior limb of the anterior commissure (IPAC), plus a few scattered neurons in the cortical amygdalar area. Only Case 7209, which extended furthest caudally into the parabrachial nucleus, contained substantial retrograde labeling in the central nucleus of the amygdala (Figure 3b). Cases 7213, 7216, and 7220 did not have any labeling in the central nucleus, and 7220 had no labeling in the amygdala, IPAC, or BST.

#### 3.1.3 Hypothalamus

The medial preoptic area contained a few scattered neurons in Cases 7209, 7213, and 7216, but none in 7220. While all cases had CTb‐labeled neurons in the paraventricular hypothalamic nucleus, Case 7209 had the most. We found light labeling scattered across several other hypothalamic nuclei (including lateral, dorsomedial, arcuate, and ventromedial), more in Case 7209 than in others. Case 7209 had many CTb‐labeled neurons in the parasubthalamic nucleus, which also had light to moderate labeling in Cases 7213, 7216, and 7220.

Although anterograde labeling was not a focus of our study, the pattern in Case 7213 was remarkable for prominent axonal CTb labeling in the dorsomedial part of the ventromedial hypothalamic nucleus (Figure 2d), reflecting the previously identified heavy projection from the superior lateral parabrachial subnucleus (Bester et al. 1997; Fulwiler and Saper 1984; Huang et al. 2020; Inagaki et al. 1984; Karthik et al. 2022). Cases 7209 and 7216 had light labeling here, but 7220 lacked anterograde labeling in the ventromedial nucleus.

#### 3.1.4 Thalamus

**FIGURE 4:**
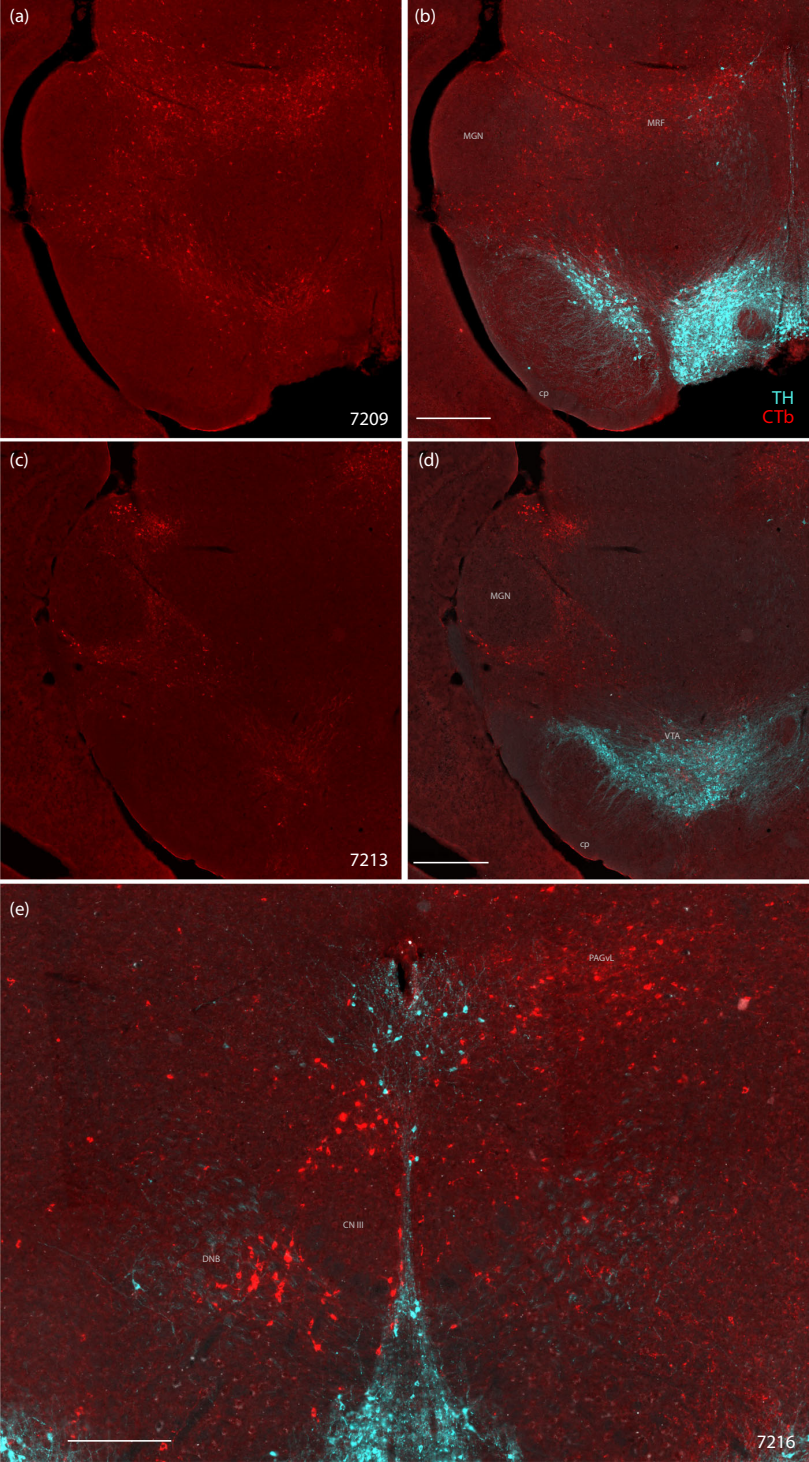
CTb labeling in the midbrain. CTb‐labeled neurons (red) in the midbrain reticular formation in (a, b) Case 7209 and (c, d) Case 7213, along with tyrosine hydroxylase (TH) immunoreactivity (ice blue) in (b) and (d). (e) CTb‐labeled neurons in Case 7216 surrounding the oculomotor nucleus (CN III), including a cluster intermingling with TH‐immunoreactive axons of the dorsal noradrenergic bundle (DNB), within the medial aspect of the central tegmental tract. The scale bar in (b) is 500 µm and applies to (a). The scale bar in (d) is 500 µm and applies to (c). The scale bar in (e) is 250 µm. cp, cerebral peduncle; MGN, medial geniculate nucleus; PAGvL, ventrolateral periaqueductal gray matter.

The medial geniculate nucleus was surrounded by many retrogradely labeled neurons in Case 7209, with moderate labeling in 7213 (Figure 4a–d) and successively fewer retrogradely labeled neurons in Cases 7216 and 7220. In Cases 7213, 7216, and 7220, but not 7209, we found a small cluster of retrogradely labeled neurons in the caudal paramedian thalamus, near the A11 group of dopaminergic neurons, in the location of the magnocellular subparafasicular thalamic nucleus (Figure 2d). Case 7213 had CTb‐labeled neurons in the posterior limitans nucleus. In addition, the pretectal region contained labeling in each case.

#### 3.1.5 Midbrain

Case 7220 had dense CTb labeling in both the deep and superficial layers of the superior colliculus. This region also contained many CTb‐labeled neurons in Case 7216, largely on the ipsilateral side. In comparison, Cases 7209 and 7213 had a moderate amount of labeling in the superior colliculus.

In every case, there were many CTb‐labeled neurons in the periaqueductal gray (PAG), but the distribution differed between cases. CTb‐labeled neurons were distributed in the ventrolateral PAG column in Case 7209, the dorsolateral PAG column in Case 7213, and both the dorsal and ventrolateral PAG columns in Case 7216. Case 7220 had many CTb‐labeled neurons in the dorsomedial and dorsolateral PAG columns (neighboring the similarly dense labeling in the overlying superior colliculus), with fewer in the ventrolateral column.

In the ventral midbrain, a moderate number of CTb‐labeled neurons spanned the retrorubral field, substantia nigra pars compacta, and ventral tegmental area in Case 7209, fewer in Case 7213, and none in Cases 7216 and 7220. While many CTb‐labeled neurons were intermingled with dopaminergic neurons in this region, none of them were immunoreactive for the dopaminergic neuronal marker tyrosine hydroxylase (Figure 4a–d).

Each case had a different pattern of retrograde labeling in the midbrain reticular formation. Case 7209 had many CTb‐labeled neurons spread throughout the midbrain reticular formation surrounding the red nucleus and extending along the dorsal aspect of the substantia nigra and ventral tegmental area. Case 7216 had scattered labeling in the midbrain reticular formation and prominent labeling surrounding the contralateral oculomotor nucleus, including a cluster of CTb‐labeled neurons intermingling with TH‐immunoreactive axons (dorsal noradrenergic bundle) along the medial aspect of the central tegmental tract (Figure 4e).

**FIGURE 5:**
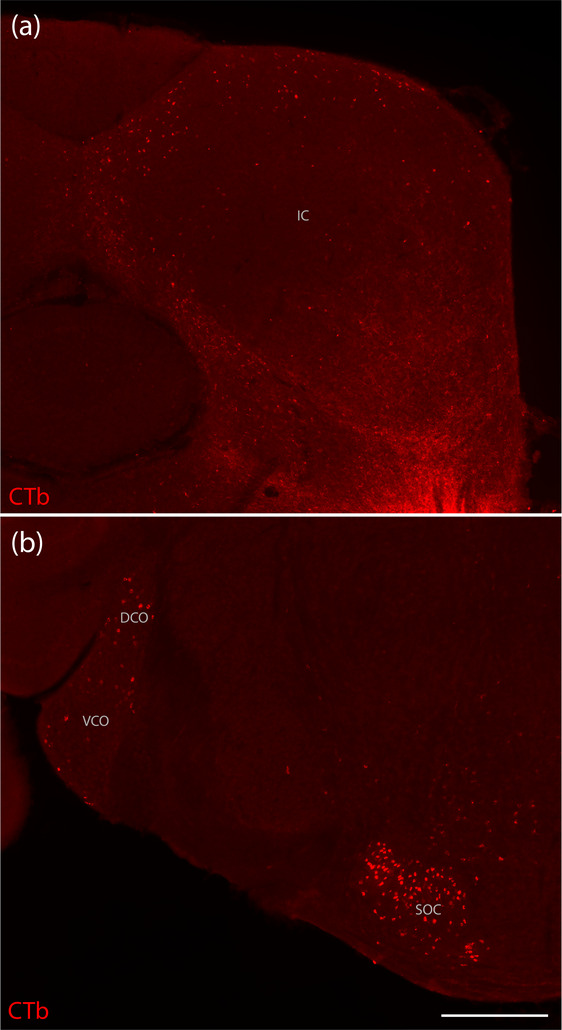
CTb labeling in auditory brainstem regions. (a) CTb‐labeled neurons (red) in the ipsilateral inferior colliculus (IC) of Case 7216. (b) CTb‐labeled neurons in the contralateral dorsal and ventral cochlear nuclei (DCO, VCO) and in the superior olivary complex (SOC) of Case 7216. The scale bar in (b) is 500 µm and applies to (a).

In the caudal midbrain, every case had retrograde labeling in the inferior colliculus (Figure 5a), with a wide range of labeling density and intensity across cases. Retrograde labeling here was light in Case 7209 and very dense in 7220, with intermediate labeling densities in 7213 and 7216. In each case, retrogradely labeled neurons were distributed primarily in the dorsal and external cortex, outside the central nucleus of the inferior colliculus.

#### 3.1.6 Hindbrain

The contralateral cochlear nucleus contained many CTb‐labeled neurons in Case 7220, and we found moderately dense labeling here in Cases 7213 and 7216 (Figure 5b) but none in 7209. The superior olivary complex had dense and predominantly ipsilateral retrograde labeling in Case 7220, bilateral labeling in Cases 7213 and 7216 (Figure 5b), and none in 7209.

A moderate number of CTb‐labeled neurons were found rostral and dorsolateral to the facial motor nucleus, flanking cranial nerve VII, in the pontine reticular formation of Case 7209. In contrast, Case 7216 contained prominent anterograde labeling in the facial motor nucleus and scattered neurons in the pontine reticular formation. Cases 7213 and 7220 had very few neurons in this region.

The hindbrain reticular formation contained a moderate number of scattered, CTb‐labeled neurons in Cases 7209 and 7216 but none in Cases 7213 and 7220. Cases 7216 and 7209 also had many CTb‐labeled neurons in the nucleus of the solitary tract and area postrema, which contained only a few neurons in Cases 7220 and 7213. The spinal trigeminal nucleus contained a moderate number of CTb‐retrogradely labeled neurons in Cases 7209 and 7216, with fewer in 7213 and only sparse labeling in 7220.

Case 7216 had a prominent concentration of CTb‐labeled neurons in the ventrolateral medulla, most of which were mutually exclusive with adjoining tyrosine–hydroxylase–immunoreactive C1–A1 noradrenergic neurons in this region. This region contained fewer, scattered neurons in Cases 7209 and 7213. Case 7220 had no labeling in the caudal ventrolateral medulla and instead had a smaller cluster of CTb‐labeled neurons between the inferior olive and the rostral ventrolateral medulla.

### 3.2 Rabies Cases

**FIGURE 6:**
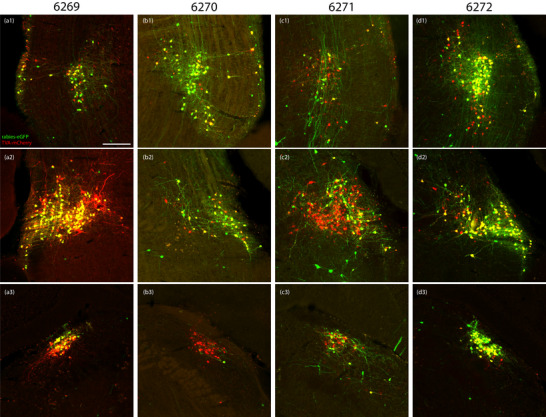
TVA‐mCherry and rabies‐eGFP injection sites. TVA‐mCherry (red), rabies‐eGFP (green), and co‐labeled “starter cells” (yellow) in Cases 6269 (a1–a3, left), 6270 (b1–b3, right), 6271 (c1–c3, right), and 6272 (d1–d3, right). The contralateral (left) injection site in bilateral Case 6271 is not shown; the other three cases did not have any starter cells on the contralateral side. The scale bar in (a1) is 200 µm and applies to all panels.

We targeted the rostral parabrachial region bilaterally in five *Nps*‐2A‐Cre mice. The distribution of “starter cells” co‐expressing TVA‐mCherry and rabies‐eGFP was unilateral in three cases (6269, 6270, and 6272) and bilateral in two (6271 and 6273). Table 5 shows cell counts, and Figures 6 (6269–6272) and 7 (6273) show TVA‐mCherry and rabies‐eGFP labeling within the injection sites from each case. Unilateral Case 6269 had starter cells and neurons expressing TVA‐mCherry alone on the left side only. Unilateral Cases 6270 and 6272 had TVA‐mCherry‐expressing neurons on both sides but rabies starter cells on the right only.

**TABLE 5:** Cell counts from the injection site in every rabies case.

Case	Side	TVA‐mCherry	Rabies‐eGFP starter cells	Percentage	Total starter cells
6269	Left	258	189	73%	189
	Right	0	0	0%	
6270	Left	324	0	0%	138
	Right	230	138	60%	
6271	Left	184	75	34.5%	159
	Right	310	84	27%	
6272	Left	386	0	0%	190
	Right	250	190	76%	
6273	Left	238	184	77%	302
	Right	201	118	58%	

*Note:* Neurons expressing TVA‐mCherry and co‐expressing rabies‐eGFP with TVA‐mCherry (starter cells) were counted in every rostrocaudal tissue section containing these markers, including but not limited to the sections shown in Figures 6 and 7.

**FIGURE 7:**
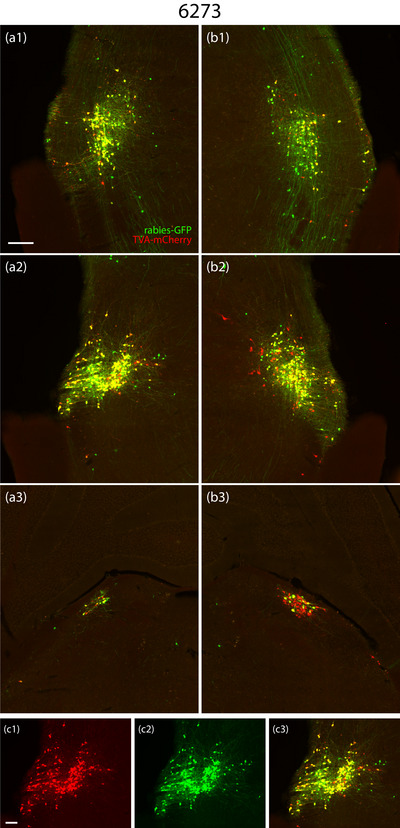
TVA‐mCherry and rabies‐eGFP injection sites in Case 6273. TVA‐mCherry (red), rabies‐eGFP (green), and co‐labeled “starter cells” (yellow) on both sides of the brain at three rostrocaudal levels in bilateral Case 6273: a rostral section including the semilunar nucleus on the left (a1) and right (b1); a middle section including the extreme lateral parabrachial nucleus on the left (a2) and right (b2); and a caudal section including the dorsolateral parabrachial nucleus on the left (a3) and right (b3). Color separation showing TVA‐mCherry (c1), rabies‐eGFP (c2), and both (c3) from panel (a2). The scale bar is 200 µm in (a1) and applies to (a2–a3, b1–b3). The scale bar is 100 µm in (c1) and applies to (c2–3).

**FIGURE 8:**
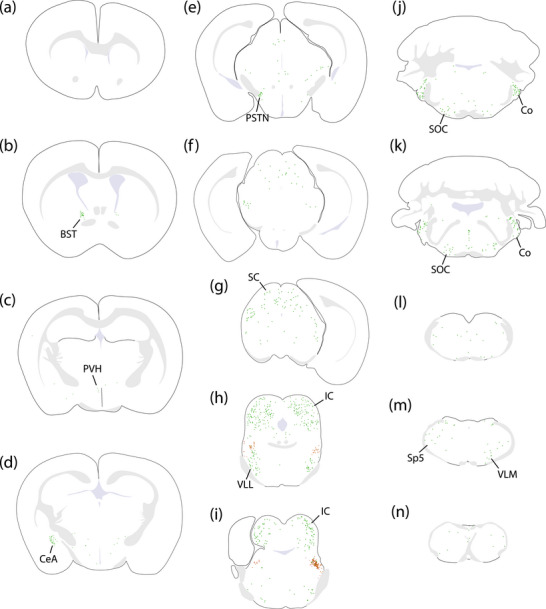
Rabies‐eGFP retrograde labeling in Case 6273. Each green dot represents a rabies‐eGFP‐expressing neuron. In panels (h) and (i), “starter cells” expressing both TVA‐mCherry and rabies‐eGFP are shown as a red ring around a green dot, and neurons expressing only TVA‐mCherry are shown as light‐red dots. BST, bed nucleus of the stria terminalis; CeA, central nucleus of the amygdala; Co, cochlear nucleus; IC, inferior colliculus; PSTN, parasubthalamic nucleus; PVH, paraventricular hypothalamic nucleus; SC, superior colliculus; SOC, superior olivary complex; Sp5, spinal trigeminal nucleus; VLL, ventral nucleus of the lateral lemniscus; VLM, ventrolateral medulla.

Across cases, the pattern of retrograde rabies‐eGFP labeling was highly similar, without any qualitative differences between unilateral left‐ (6269) and right‐sided (6270 and 6272) cases. No left‐right difference was apparent in the labeling pattern between the bilateral cases (6271 and 6273). We selected Case 6273, which had the most rabies starter cells bilaterally, as the representative case to display this common brain‐wide labeling pattern (Figure 8).

#### 3.2.1 Cerebral Cortex

**FIGURE 9:**
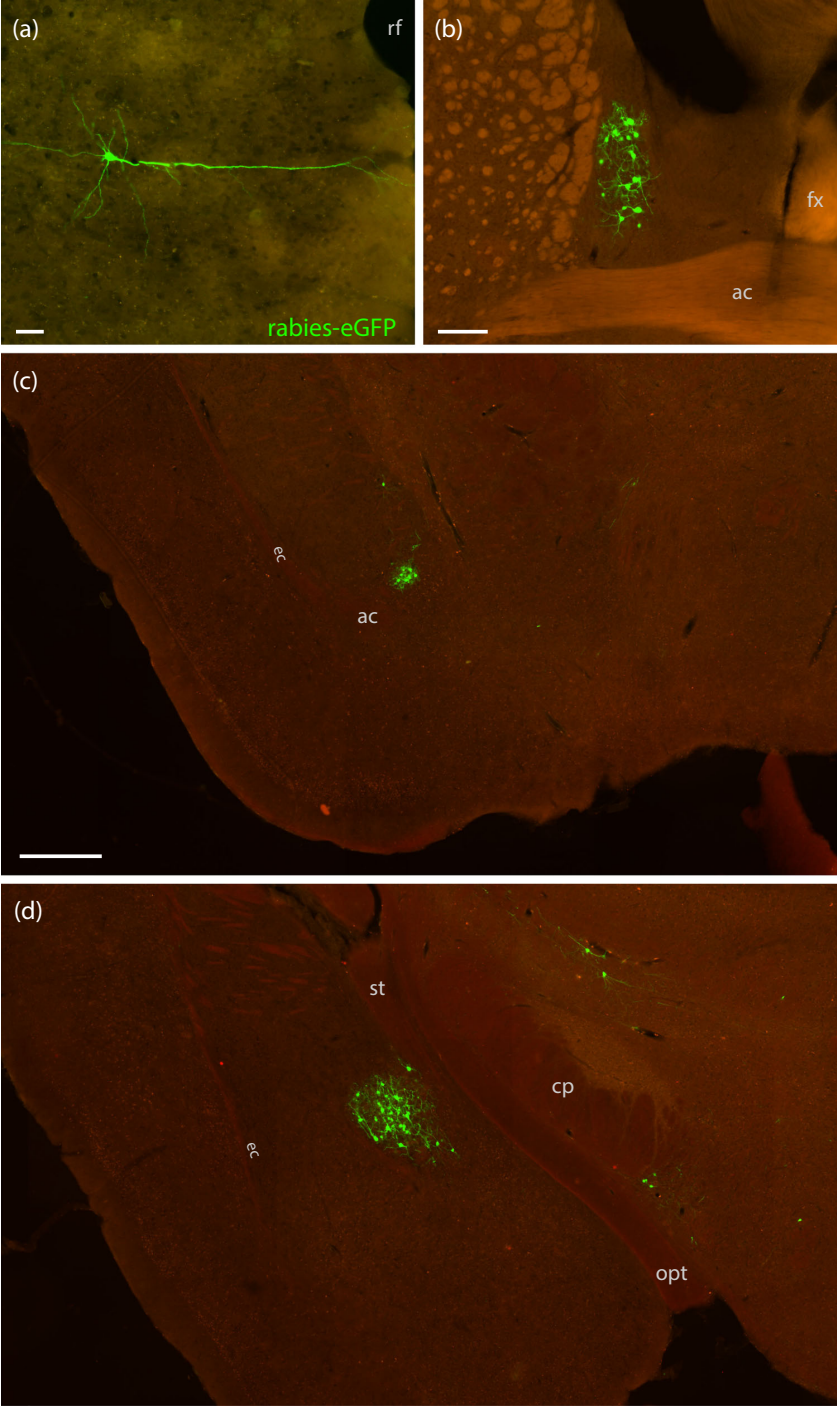
Rabies‐eGFP labeling in the cerebral cortex and extended amygdala. (a) A solitary rabies‐eGFP‐labeled (green) pyramidal neuron in the ectorhinal/perirhinal cortical area in Case 6271. (b) Rabies‐eGFP‐labeled neurons in the bed nucleus of the stria terminalis in Case 6270. (c) Cluster of rabies‐eGFP‐labeled neurons in the interstitial nucleus of the posterior limb of the internal capsule (IPAC) and (d) central nucleus of the amygdala in Case 6273. The scale bar in (a) is 50 µm. The scale bar in (b) is 200 µm. The scale bar in (c) is 400 µm and applies to (d). ac, anterior commissure; cp, cerebral peduncle; ec, external capsule; fx, fornix; opt, optic tract; rf, rhinal fissure; st, stria terminalis.

In contrast to the copious cortical labeling in every CTb case, only scattered neurons in the cerebral cortex expressed rabies‐eGFP. The rare, individual neurons retrogradely infected with rabies‐eGFP were scattered among motor, somatosensory, auditory, and perirhinal cortices. Except for one small neuron in Layer 6 of the insular cortex (Case 6271), all rabies‐eGFP‐labeled neurons in the cortex were located in Layer 5 and had a pyramidal morphology with a prominent apical dendrite (Figure 9a).

#### 3.2.2 Bed Nucleus of the Stria Terminalis

The bed nucleus of the stria terminalis contained rabies‐eGFP‐labeled neurons in every case. Virtually all of these neurons were located in the oval subnucleus (Figure 9b), but we found an individual neuron ventral to the anterior commissure in three cases.

#### 3.2.3 Amygdala

The interstitial nucleus of the posterior limb of the anterior commissure (IPAC) contained a dense cluster of rabies‐eGFP retrogradely labeled neurons in every case (Figure 9c). Caudally, we found a larger group of rabies‐eGFP‐labeled neurons in the central nucleus of the amygdala, primarily in its lateral subdivision, with occasional neurons in the capsular and medial subdivisions (Figure 9d).

#### 3.2.4 Hypothalamus

No cases had prominent labeling in the hypothalamus. Rostrally, a few neurons were found in the preoptic area, and each case contained a small number of neurons in the paraventricular hypothalamic nucleus. In addition to a handful of neurons scattered across the lateral hypothalamic area, each case had a small collection of neurons in the parasubthalamic nucleus and in the overlying zona incerta.

#### 3.2.5 Thalamus

Except for a small number of neurons in pretectal and peri‐geniculate regions, the thalamus contained virtually no rabies‐eGFP‐labeled neurons.

#### 3.2.6 Midbrain

**FIGURE 10:**
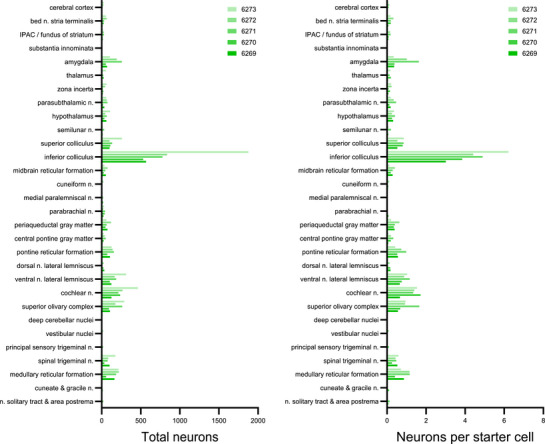
Total counts of rabies‐eGFP‐labeled neurons and per starter cell. Separated by brain region and case number.

**FIGURE 11:**
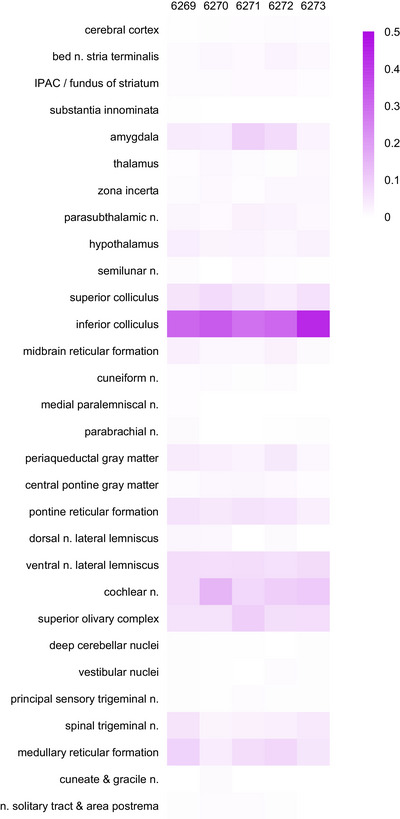
Relative proportion of rabies‐eGFP‐labeled neurons by brain region. Heatmap showing rabies‐eGFP‐labeled neurons across brain regions as a proportion of total rabies‐eGFP‐labeled neurons in each case.

**FIGURE 12:**
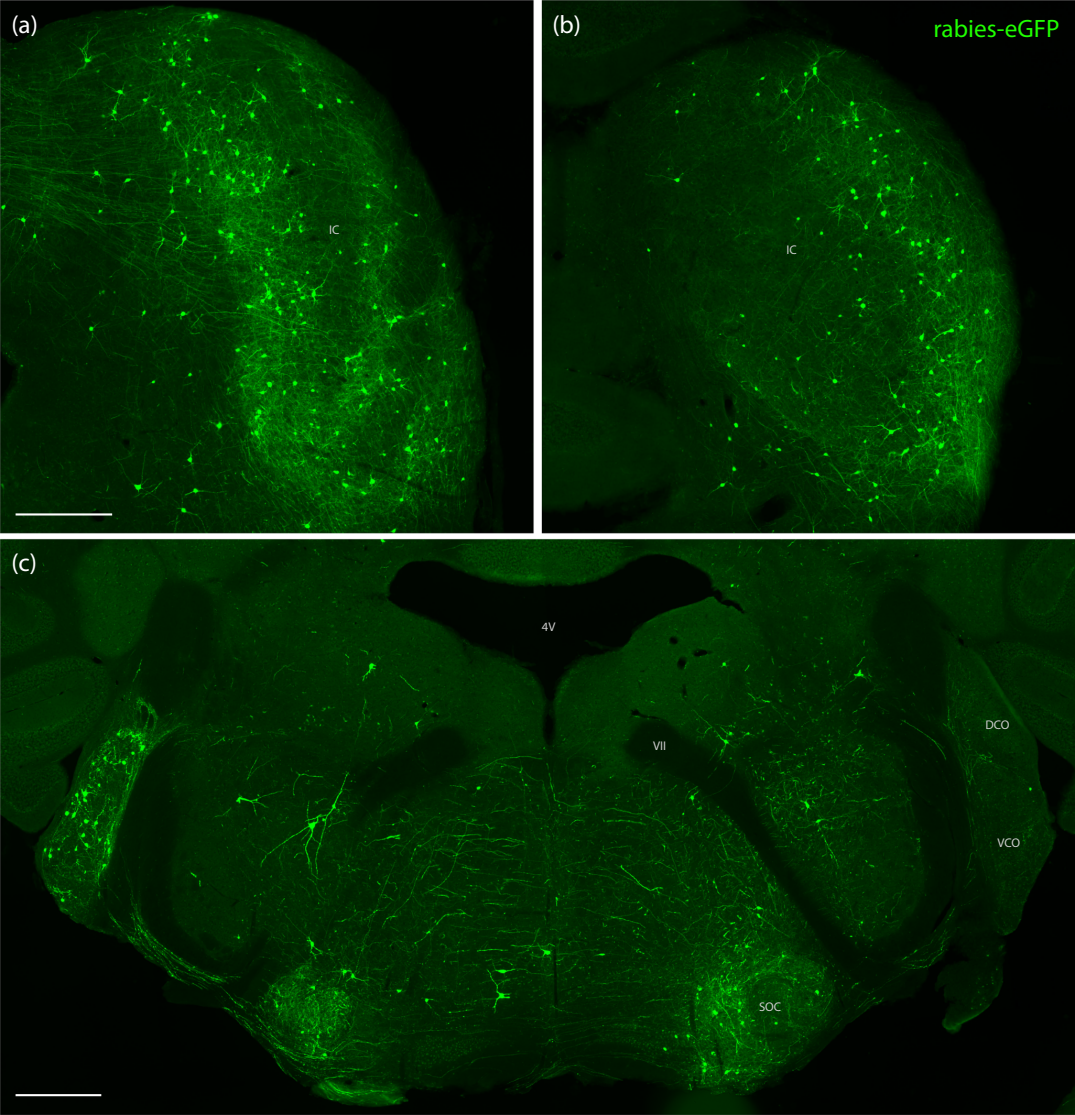
Rabies‐eGFP labeling in auditory brainstem structures. Rabies‐eGFP (green) labeling pattern in the rostral (a) and caudal (b) inferior colliculus (IC) in Case 6273. (c) Rabies‐eGFP‐labeled neurons in the dorsal and ventral cochlear nuclei (DCO and VCO), superior olivary complex (SOC), and reticular formation in Case 6272. The scale bar in (a) is 400 µm and applies to (b). The scale bar in (c) is 500 µm. 4V, fourth ventricle; VII, facial nerve genu.

Auditory‐related regions of the midbrain contained the greatest numbers of rabies‐eGFP‐labeled neurons in every case, measured by their total number, number per starter cell (Figure 10), or as a proportion ofthe total number in each brain (Figure 11). In particular, the inferior colliculi contained the greatest number of rabies‐eGFP‐labeled neurons in every case. In the rostral inferior colliculus, rabies‐eGFP labeling appeared to concentrate in three layers, forming a tulip shape (Figure 12a). Caudally, labeled neurons avoided the central nucleus and concentrated mostly in the lateral cortex of the inferior colliculus (Figure 12b). The ventral nucleus of the lateral lemniscus also contained dense rabies‐eGFP labeling in every case.

Rabies‐eGFP‐labeled neurons in the PAG were predominantly concentrated in the ventrolateral column. The midbrain reticularformation contained scattered rabies‐eGFP‐labeled neurons. Most of these neurons, especially rabies‐eGFP‐labeled neurons in the pontine and medullary reticular formation, had long, prominent dendrites.

**FIGURE 13:**
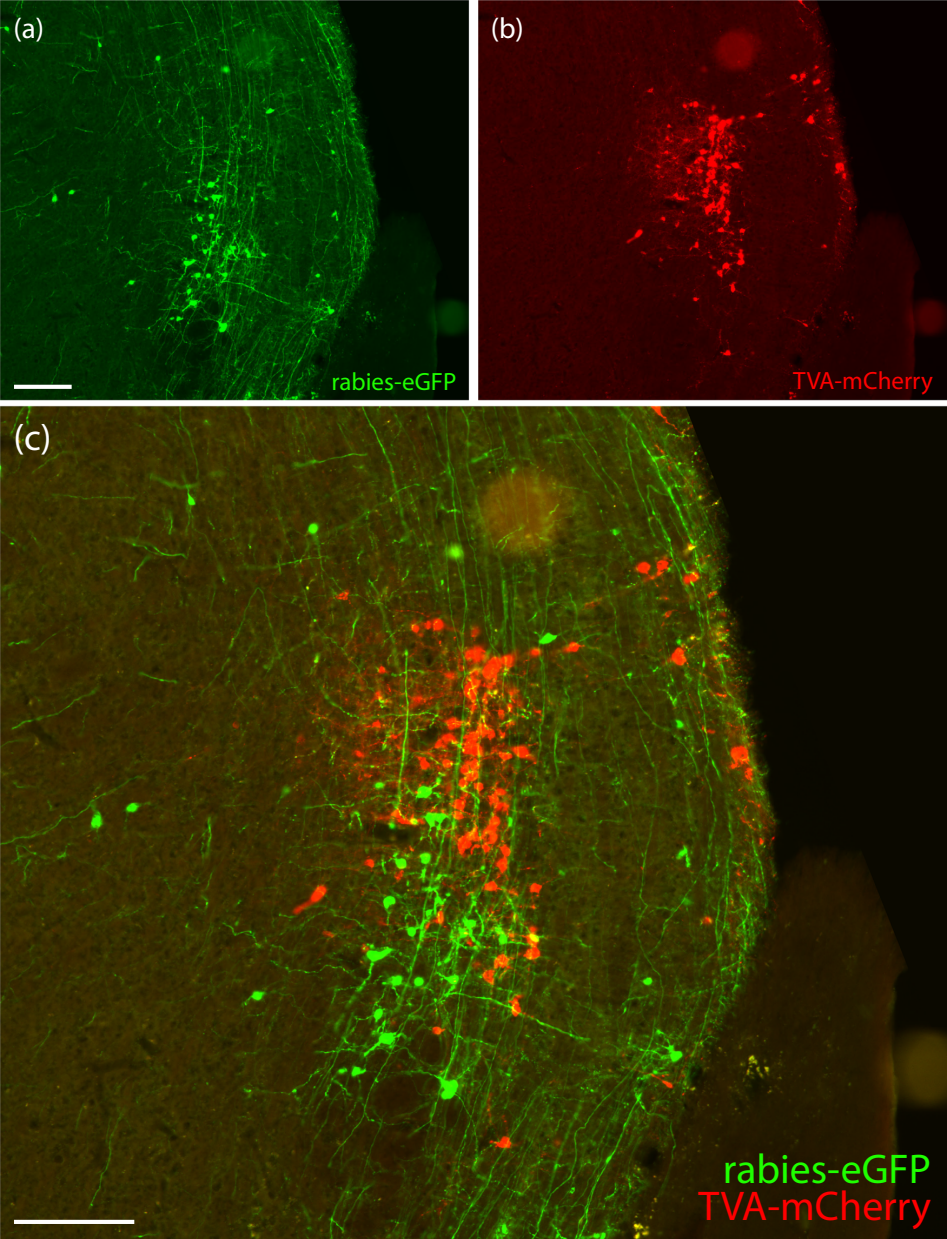
Rabies‐eGFP labeling in the medial parameniscal nucleus in Case 6271. Rabies‐eGFP‐labeled neurons (green in a, c) in the medial parameniscal nucleus distributed ventral to TVA‐mCherry‐labeled neurons (red in b, c) in the semilunar nucleus of Case 6271. The scale bar in (a) is 200 µm and applies to (b). The scale bar in (c) is 200 µm.

In the medial paralemniscal nucleus, all cases contained rabies‐eGFP‐labeled neurons that were mutually exclusive with the NPS neurons expressing TVA‐mCherry immediately dorsal to them in the semilunar nucleus. This labeling in the medial paralemniscal nucleus, which receives input from the auditory cortex and inferior colliculus (Varga et al. 2008), was most prominent in Case 6271 (Figure 13c).

#### 3.2.7 Hindbrain

Several auditory‐related regions of the hindbrain were prominently labeled with rabies‐eGFP. The dorsal and ventral cochlear nuclei contained many rabies‐eGFP‐labeled neurons. In unilateral cases, the contralateral cochlear nucleus contained the vast majority of rabies‐eGFP‐labeled neurons (Figure 12c). The superior olivary complex contained a moderate number of rabies‐eGFP‐labeled neurons; in unilateral cases, most of these neurons were ipsilateral to the rabies starter cells (Figure 12c).

The pontine reticular formation contained dispersed rabies‐eGFP‐labeled neurons in every case. A few rabies‐eGFP‐labeled neurons were also found in the central pontine gray. The principal sensory trigeminal nucleus contained a few neurons in every case, and the spinal trigeminal nucleus contained a moderate number of rabies‐eGFP‐labeled neurons. We also found many rabies‐eGFP‐labeled neurons scattered throughout the medullary reticular formation (Figure 12c). Small numbers of rabies‐eGFP‐labeled neurons were found in the nucleus of the solitary tract and area postrema, gracile nucleus, cuneate nucleus, and vestibular nucleus.

#### 3.2.8 Cerebellum

While the cerebellar cortex did not have any rabies‐labeled neurons in most cases, we found one Purkinje neuron expressing eGFP in the flocculus of Case 6269. We also found one or a few neurons scattered in the deep cerebellar nuclei of every case except 6272.

### 3.3 Anterograde Tracing From the Cerebral Cortex

**FIGURE 14:**
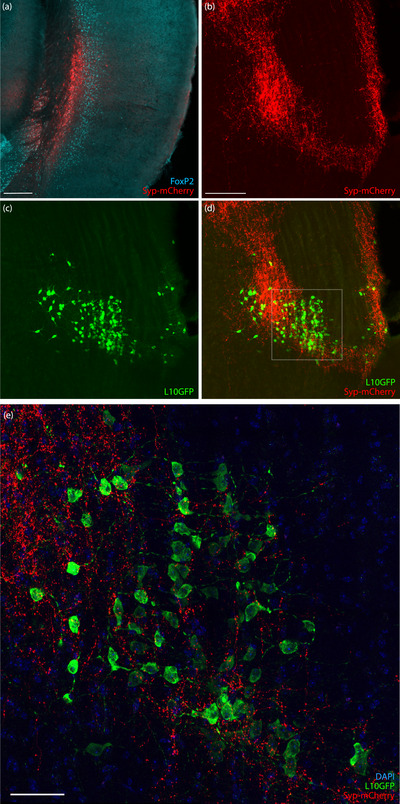
Anterograde labeling from the cerebral cortex. (a) Neurons in the injection site express Syp‐mCherry (red) in layer 6b, deep to FoxP2‐immunoreactive neurons in layer 6a (ice blue). (b) Abundant Syp‐mCherry‐labeled axons and boutons overlapped the main concentration of L10GFP‐expressing (green) NPS neurons (c, d). (e) Confocal imaging of Syp‐mCherry‐labeled boutons and L10GFP‐expressing somata and proximal dendrites with DAPI (blue). The scale bar in (a) is 250 µm. The scale bar in (b) is 250 µm and applies to (c) and (d). The scale bar in (e) is 50 µm.

A major discrepancy between CTb and rabies retrograde labeling patterns across cases was the copious CTb yet sparse rabies‐eGFP labeling in the cerebral cortex. To examine this difference, we injected AAV8‐hEf1a‐synaptophysin‐mCherry into the deep temporal association cortex, ectorhinal, and perirhinal areas in three *Nps*‐2A‐Cre;R26‐LSL‐L10GFP reporter mice. These injections transduced deep cortical neurons in Layer 6b between approximately 1.9 and 2.8 mm caudal to bregma (Figure 14a). In every case, we found punctate Syp‐mCherry labeling encompassing the main cluster of *Nps*‐expressing neurons (Figure 14d). Confocal imaging revealed many Syp‐mCherry‐labeled boutons contacting L10GFP‐expressing neuronal somata and proximal dendrites (Figure 14e).

## 4 Discussion

This paper builds upon our previous work on the molecular identity and efferent projections of NPS neurons. NPS neurons have a distinctive distribution, with a dense concentration in the extreme rostral–lateral part of the parabrachial region. From there, they extend rostrally into the semilunar nucleus and caudally into the dorsolateral parabrachial subnucleus. In contrast to the varied input patterns labeled by injections of the nonselective retrograde tracer CTb into this region, we found a consistent pattern of retrograde labeling in auditory‐related regions and in the reticular formation after Cre‐conditional rabies tracing. Below, we discuss these findings within the context of previous neuroanatomical studies then explore functional implications and examine limitations of our approach.

### 4.1 Comparison to Previous Neuroanatomical Work

Input connections to NPS neurons in the parabrachial region have been examined by two groups investigating the regulation of wakefulness (Angelakos et al. 2023; Xing et al. 2024). Similar to our approach, Angelakos et al. performed rabies retrograde tracing from the lateral parabrachial nucleus in *Nps*‐IRES‐Cre mice. They reported labeling in the bed nucleus of the stria terminalis, central amygdala, inferior colliculus, ventral cochlear nucleus, pontine reticular nucleus, and periaqueductal gray. However, they also reported that the second largest input in the brain arose from local neurons in the lateral parabrachial area (Fig. 2I of Angelakos et al. 2023). In contrast, we found very few nonstarter cells (rabies‐eGFP‐labeled neurons without TVA‐mCherry labeling) in the parabrachial region, possibly because we allowed more time for TVA‐mCherry expression, which enabled a clear distinction between starter cells (TVA‐mCherry‐ and rabies‐eGFP‐expressing) and retrogradely labeled neurons (rabies‐eGFP‐only). Based on these findings, we conclude that local interneurons are not a major source of input to parabrachial NPS neurons. With this exception, the proportions of inputs reported in that study are largely consistent with the pattern we found. Xing et al. also performed rabies retrograde tracing from the parabrachial region in *Nps*‐IRES‐Cre mice and reported inputs from the bed nucleus of the stria terminalis, central nucleus of the amygdala, and lateral hypothalamus. No injection site was shown, so the distribution of starter neurons was unclear, and the lack of labeling in auditory‐related brain regions could be due to the different helper viruses and modified rabies virus used in that study, or to their allowing only 1 week for TVA‐mCherry expression before injecting rabies.

In our CTb cases, the patterns of retrograde labeling produced by four injection sites overlapping NPS neurons differed in several respects from previously published retrograde tracing data in this region. Previous retrograde tracer injections in the rat parabrachial nucleus labeled neurons in the medial, orbitofrontal, and insular cortical areas, as well as the bed nucleus of the stria terminalis, substantia innominata, central nucleus of the amygdala, several hypothalamic nuclei, periaqueductal gray matter, ventral midbrain, hindbrain reticular formation, nucleus of the solitary tract, area postrema, and spinal trigeminal nucleus (Herbert et al. 1990; Moga et al. 1990). This pattern closely matches the pattern of retrograde labeling in our CTb case with the most caudal injection site, spreading back into the middle of the parabrachial nucleus (Case 7209).

In contrast, the retrograde labeling pattern in cases with more rostral injection sites (Cases 7213, 7216, and 7220) deviated from the previously reported parabrachial pattern. These cases had substantially less labeling in the bed nucleus of the stria terminalis and central nucleus of the amygdala and abundant labeling in auditory‐related brain regions, including the dorsal and ventral nuclei of the lateral lemniscus, cochlear nucleus, and superior olivary complex. We were initially skeptical of the likelihood that this prominent pattern of labeling in auditory‐related regions reflects input to the NPS neurons due to the possibility of CTb entering “axons of passage” traveling through the lateral lemniscus (Chen and Aston‐Jones 1995). However, prominent rabies‐eGFP labeling in these same regions supports an important role for direct auditory‐related input to NPS neurons.

In every case, input from auditory‐related brain regions comprised the plurality of rabies‐eGFP‐labeled neurons. The rabies‐eGFP pattern resembled labeling in CTb cases with more rostral injection sites, with the notable exception of the cerebral cortex. In contrast to the abundant retrograde labeling in every CTb case, we only found scattered rabies‐eGFP‐labeled neurons in the cerebral cortex. However, anterograde tracing from cortical neurons in Layer 6b of the temporal association, ectorhinal, and perirhinal areas labeled presynaptic boutons overlapping NPS neurons, with many axo‐somatic and axo‐dendritic close contacts. The sparse labeling in our rabies cases could reflect a reluctance of rabies to label cortical neurons, as reported in previous studies (Ährlund‐Richter et al. 2019; Sun et al. 2019). Channelrhodopsin‐assisted circuit mapping (Petreanu et al. 2007) in *Nps*‐2A‐Cre;R26‐LSL‐L10GFP Cre‐reporter mice could help resolve this question.

#### 4.1.1 Functional Implications

Neurons in the parabrachial nucleus relay diverse sensory information, including pain, temperature, taste, and visceral signals, to the forebrain to influence behavior (Carter et al. 2015; Chamberlin and Saper 1992; Geerling et al. 2016; Jarvie et al. 2021; Kim et al. 2020; Nakamura and Morrison 2008; Park et al. 2020). NPS neurons in this region send projections to brain regions involved in stress and threat‐response—paraventricular thalamus, dorsomedial hypothalamus, periaqueductal gray, and ventral bed nucleus of the stria terminalis—as well as regions implicated in circadian function: the subparaventricular zone, intergeniculate leaflet, and magnocellular subparafasicular nucleus (Zhang et al. 2024). Consistent with this output pattern, previous studies explored a role for NPS neurons in increasing wakefulness (Angelakos et al. 2023; Xing et al. 2024). However, the function of NPS neurons in relation to their inputs remains mysterious.

NPS neurons receive substantial auditory input, with less input from the reticular formation, bed nucleus of the stria terminalis, and central nucleus of the amygdala. The predominance of auditory‐related input was particularly striking and unexpected. This strong input from neurons low in the auditory hierarchy suggests that NPS neurons receive minimally processed acoustic information. Beyond auditory inputs, the medullary and pontine reticular formation, followed by the spinal trigeminal nucleus, were the next largest contributors. The brainstem reticular formation contains diffuse isodendritic neurons that integrate heterogeneous sensory information and help control posture, arousal, noxious reflexes, startle responses, and cardiorespiratory reflexes (French 1958; Ramón‐Moliner and Nauta 1966; Schepens and Drew 2004; Wang 2009). The spinal trigeminal nucleus receives sensory information from the face, mouth, and other parts of the head (Sessle 2000). Additional input to NPS neurons comes from the bed nucleus of the stria terminalis and central nucleus of the amygdala, which are implicated in anxiogenic states and threat responses (Davis et al. 2010; Moscarello and Penzo 2022; Walker et al. 2003).

Overall, the connectivity pattern of NPS neurons suggests that these neurons integrate anxiogenic and threat‐related information from the bed nucleus of the stria terminalis and central nucelsu of the amygdala with predominantly auditory information, supplemented by heterogeneous sensory information from the reticular formation and spinal trigeminal nucleus. This positions NPS neurons to assess and process auditory information in relation to threat and anxiety calculations and in the context of physiologically salient information. NPS neurons then relay this integrated information to forebrain areas that coordinate behavioral responses. Brain regions implicated in auditory fear conditioning include the auditory cortex, medial geniculate nucleus, lateral amygdala, and inferior colliculus (Boatman and Kim 2006). Notably, NPS neurons receive substantial input from the inferior colliculus, but there is limited connectivity with the medial geniculate nucleus and lateral amygdala. A previous study examining the auditory startle response in NPSR1‐deficient mice found a significant difference in the startle response in NPSR1 knockout mice compared to wild‐type mice (Fendt et al. 2011). Clinically, a human genome‐wide association study found a single‐nucleotide polymorphism (SNP) in the *NPS* gene linked to migraineurs (de Marco et al. 2026), so it is tempting to speculate a possible role for NPS neurons in phonophobia associated with migraine headaches. However, the specific role of NPS neurons in auditory processing and behavioral responses remains unknown.

### 4.2 Limitations

The Cre‐conditional retrograde tracing approach we used begins with Cre‐dependent expression of TVA‐mCherry and rabies G protein. Once activated by Cre, their expression and subsequent rabies‐eGFP labeling are unrelated to the varying levels of *Nps* expression in a given cell. This is a relevant consideration because NPS neurons in the main cluster within the extreme lateral parabrachial nucleus have consistently high levels of *Nps* mRNA and NPS immunoreactivity, while neurons in the semilunar nucleus and parabrachial dorsal lateral subnucleus express less *Nps* mRNA and often lack NPS immunoreactivity under baseline conditions (Huang et al. 2022). Rabies starter cells covered a wide distribution of *Nps*‐expressing neurons in this region in our combined experiments, and retrograde labeling with rabies‐eGFP presumably represents their combined total set of afferents. Exploring the possibility that distinct parabrachial and semilunar subpopulations have distinct sets of afferent connections would require specific genetic markers to distinguish between them.

In addition, rabies has decreased avidity for specific neuron types. After injection sites centered in the lateral hypothalamic area, proportionally fewer neurons in the cerebral cortex were labeled by modified rabies than by retro‐AAV (Sun et al. 2019). Rabies also may not label *Sst*‐expressing interneurons in the prefrontal cortex, nonpeptidergic neurons in dorsal root ganglia, and parabrachial‐projecting neurons in the caudal nucleus of the solitary tract (Ährlund‐Richter et al. 2019; Albisetti et al. 2017; Korkutata et al. 2025). Finally, because this technique produces equally robust eGFP expression in every rabies‐infected neuron, it is not useful for determining the relative strength of each input. Future anterograde tracing and channelrhodopsin‐assisted circuit mapping experiments could address these limitations and compare the functional strengths of individual connections.

Another limitation of our study is that it was not designed to evaluate potential sex differences in neuroanatomical connectivity. Evidence exists for differences in brain volume (Meyer et al. 2017) and connectivity (Hutton et al. 1998) between male and female mice, primarily in the hypothalamus. No studies examining the neurons that produce NPS have identified a difference in connectivity between male and female animals, but given previously documented differences in the hypothalamus, future studies specifically designed to examine sex as a biological variable could address this limitation.

### 4.3 Conclusion

We used conventional retrograde axonal tracing to identify the brain‐wide pattern of afferents to the extreme rostral–lateral parabrachial region, which contains the densest concentration of NPS neurons. This approach and Cre‐dependent rabies retrograde tracing identified heavy input from auditory brainstem structures. We also used anterograde tracing to examine afund discrepancy in cortical labeling between conventional and rabies approaches and found many close contacts between cortical boutons and *Nps‐*expressing neurons. These findings reveal an unexpected role for NPS neurons as integrators of auditory information.

## Authors Contributions

J.C.G. planned the project and secured funding. R.Z. and J.C.G. drafted and edited the manuscript. R.Z. and S.G. performed stereotaxic injections, histology, and microscopy. S.G. performed confocal microscopy. R.Z. and J.C.G. drafted and edited the figures and figure legends.

## Ethics Statement

All procedures performed in studies involving animals were in accordance with the ethical standards of the institution or practice at which the studies were conducted.

## Conflicts of Interest

The authors declare no conflicts of interest.

## Data Availability

The data that support the findings of this study are available from the corresponding author upon reasonable request.
